# Anterior cruciate ligament reconstruction with a biocomposite interference screw maintains graft fixation survival and improves clinical outcomes at 1 year: A multicenter prospective case series

**DOI:** 10.1016/j.heliyon.2023.e20921

**Published:** 2023-10-12

**Authors:** Anup Shah, Geoffrey Van Thiel

**Affiliations:** a Orthopedic Surgery, Banner University Medical Group, Arizona, United States; b Orthopedic Surgery-Sports Medicine, OrthoIllinois, Illinois, United States

## Abstract

A biocomposite interference screw with an open architecture was developed to provide a greater available surface area for bone ingrowth compared with conventional solid interference screws. We herein describe a prospective, single-cohort study conducted using this interference screw in anterior cruciate ligament (ACL) reconstruction. Sixty subjects (mean age, 34.9 years; standard deviation, 10.6) undergoing ACL repair using the biocomposite interference screw were enrolled at 3 sites in the United States. Subjects were followed preoperatively (baseline) and postoperatively at 6 months and 1 year. The primary endpoint was graft fixation survival rate at 6 months and 1 year. Secondary endpoints included graft survival (failure defined as that occurring for any reason); International Knee Documentation Committee (IKDC) score (exam and subjective forms); Tegner Activity form; Lysholm score; and EQ (EuroQuol)-5D-5L index score and visual analogue scale. There was a 100 % rate of graft fixation survival at 6 months (54/54; 95 % confidence interval [CI]: 100-100) and 1 year (50/50; 95 % CI: 100-100). One patient experienced a complete tear of the ACL 5 months following index surgery, resulting in graft survival rates of 98.1 % (53/54; 95 % CI: 94.6–100) at 6 months at 98.0 % (49/50; 95 % CI: 94.1–100) at 1 year. Significant improvements (p < .0001) were obtained between baseline and 6 months for the majority of patient-reported outcomes, and were maintained out to 1-year follow up. There was no significant difference over baseline in the IKDC sub-scale of symptoms. Nine patients (15.0 %) experienced serious adverse events during the course of the study; three of these patients’ adverse events were considered possibly or definitely related to the procedure device (ACL tear, pulmonary embolism/deep vein thrombosis, and a patellar fracture). In conclusion, this biocomposite interference screw has a favorable safety and efficacy profile at 1 year, with no failures of graft fixation, noted during that time.

## 1 Introduction

Isolated tears of the anterior cruciate ligament (ACL) are a commonly encountered orthopedic injury, estimated to occur at a rate of 68.6 per 100,000 person-years [1]. ACL tears are associated with significant short- and long-term morbidity, including chronic pain, impaired function, decreased quality of life, and increased risk of developing early-onset osteoarthritis [2].

Because damage to the local articular cartilage following ACL tears impairs their ability to heal biologically, many surgeons decide to reconstruct the ligament using natural grafts [3]. Considered the gold-standard treatment in this indication [3], ACL reconstruction aims to restore stability and function to the knee, while preventing further damage. It also may confer protective effects on the meniscus [4]. ACL reconstructions are a more cost-effective approach than structured rehabilitation [5]. For these reasons, ACL reconstructions are an increasingly performed surgery, rising in prevalence by 37 % between 1994 and 2006 in the United States [6].

Interference screws are a popular method among surgeons for securing the ACL graft to obtain adequate fixation during the early postoperative and rehabilitation period [7]. Bioabsorbable screws have several proposed advantages over their titanium counterparts, including reduced risk of graft damage during insertion, achieving reduced stress shielding via gradual load transfer throughout the degradation process, lack of distortion upon magnetic resonance imaging (MRI), and the superior conditions for revision surgery, should it be required, afforded by implant's ability to be drilled through in the short-term postoperative period and eventual absorption and replacement by bone later on [8]. These characteristics are important to consider due to their effect on device safety and efficacy, and ultimately, success of the reconstruction.

A biocomposite interference screw with an open architecture was developed to provide a greater available surface area for bone ingrowth compared with conventional solid interference screws. In addition, the open architecture imparts circumferential graft-to-bone integration. Collectively, these features may improve graft fixation and clinical outcomes in ACL reconstruction by mitigating risk of graft slippage. We herein describe a prospective, single-cohort, multicenter study conducted using this interference screw in ACL reconstruction. The objective of the study was to provide safety and performance data with the device during the initial 1-year postoperative period, with a focus on its ability to promote graft fixation.

## 2 Methods

### 2.1 Participant recruitment

Between January 2018 and August 2019, investigators at 3 sites in the United States enrolled adult subjects (aged ≥18 years) undergoing ACL reconstruction using the biocomposite interference screw. Eligible patients were willing and able to comply with the study visit schedule and to complete study procedures and questionnaires.

Patients were excluded if they met any of the following criteria: a body mass index >40; total knee arthroplasty in the study knee; conditions that may interfere with graft survival or outcome (e.g. Paget disease, vascular insufficiency, muscular atrophy, uncontrolled diabetes, moderate-to-severe renal insufficiency or neuromuscular disease); a known allergy to the study device or any of its components; pregnant, or plans to become pregnant during the study; an emotional or neurological condition that would pre-empt their willingness to participate in the study; entered in another investigational drug, biologic, or device study, or has been treated with an investigational product in the past 30 days; and/or known to be at risk for loss to follow-up or failure to return for scheduled visits.

Institutional review board approval (Western Institutional Review Board, Puyallup, Washington) was obtained for each investigational site, and the study was performed in compliance with the Declaration of Helsinki. All patients provided voluntary informed written consent before enrollment. This trial was registered at ClinicalTrials.gov (NCT03519555).

### 2.2 Study outcomes

Relevant demographic information, medical history, and baseline clinical outcomes were obtained preoperatively. Patients were followed up at 6 months and 1 year postoperatively.

The primary endpoint was graft fixation survival rate at 6 months and 1 year. Graft reconstruction failure was diagnosed using the Lachman test, with the examiner grasping the tibia at the level of the tibial tubercle while stabilizing the femur with the other hand. The patient relaxes the leg while the examiner holds the knee flexed at 25°–30° and pulls forward on the tibia while stabilizing the femur. Excessive motion relative to the opposite knee or a soft endpoint of greater than 4 mm displacement is positive (i.e. abnormal). In the event of suspected graft reconstruction failure, MRI was conducted to confirm the root cause of the failure (i.e. intraarticular graft failure or fixation failure).

Secondary endpoints included graft survival at 6 months and 1 year (failure defined as that occurring for any reason) as well as the following patient-reported outcomes: International Knee Documentation Committee (IKDC) score (exam and subjective forms); Tegner Activity form; Lysholm score; EQ (EuroQuol)-5D-5L index score and visual analogue scale (VAS).

Finally, serious adverse events and adverse device effects occurring intra-operatively and postoperatively were recorded.

### 2.3 Study device and surgical technique

All patients underwent ACL reconstruction with grafts secured using the same bioabsorbable interference screw (BIOSURE REGENESORB™; Smith + Nephew, Andover, MA, USA), in accordance with the manufacturer's instructions for use. The screw's biocomposite material comprises the copolymer poly (l-lactide co-glycolide; PLGA) in combination with calcium sulfate and beta-tricalcium phosphate (β-TCP), which have demonstrated osteoconductive mechanisms of action [[9], [10], [11], [12]]. β-TCP facilitates bone formation over 18 months [9] and provides the scaffolding for subsequent bone formation [10]. Calcium also contributes to the enhanced distribution of local growth factors [13]. The implant is designed to remain stable for a minimum of 6 months before eventually being absorbed within the first 2 postoperative years.

### 2.4 Statistical analysis

This study did not include a formal sample size calculation, as there are no previous studies with the implant or relevant literature to use as a basis for this. Due to this lack of prior information, a sample size of 60 subjects was recruited for this study based on feasibility only.

If any individual IKDC, Tegner, Lysholm or EQ-5D-5L item responses were missing at a postbaseline visit, then the data were input from the previous assessment using the Last Observation Carried Forward (LOCF) method. If baseline was the only data point available, then no imputation methods were used, and baseline data were not carried forward. If all responses were missing at a post-baseline visit (including if a subject has discontinued from the study), then all missing scores were carried forward using the LOCF method as above, with no carrying forward of baseline values.

Unless otherwise stated, all significance tests and hypothesis testing are two-sided and performed at the 5 % significance level. Where data summaries are specified, categorical and ordinal variables are summarized using frequency distributions detailing the number and percentage of subjects that fall into each category. Continuous variables are summarized using the following summary statistics: mean, standard deviation, minimum and maximum values, and number of observations. In formal analyses, resulting p-values are quoted, with statistical significance set at p < .05, and 95 % two-sided confidence intervals (CIs) generated where appropriate. All statistical calculations were made using SAS software version 9.4 (SAS Institute, Cary, NC, USA).

## 3 Results

**Fig. 1:**
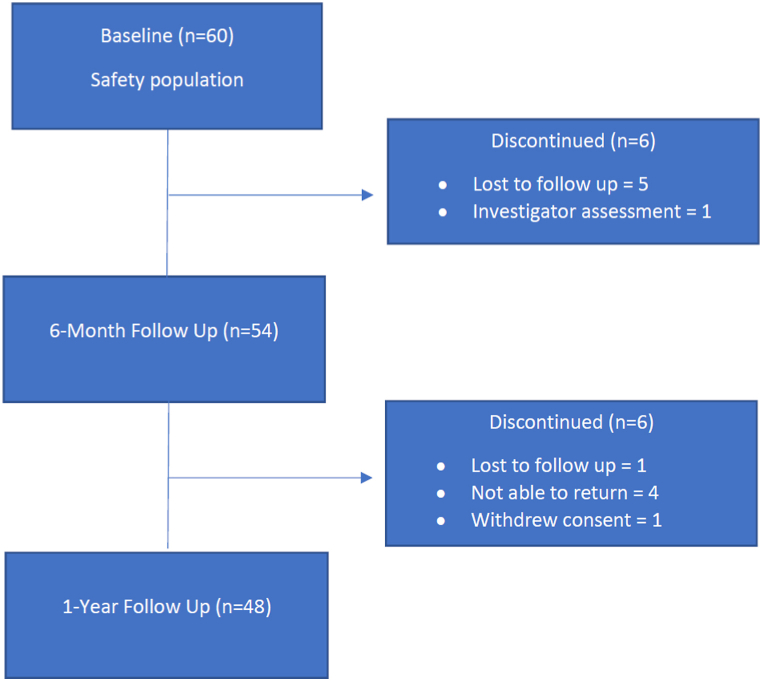
Study flow diagram showing number of study participants at 6-month and 1-year follow up and reasons for discontinuation.

Sixty subjects were enrolled and underwent arthroscopic single-tunnel ACL reconstruction using the study device (Table 1). Information regarding patient discontinuation and participation at follow-up visits is provided in Fig. 1.

**Table 1:** Patient demographics and operative data (n = 60).

Demographics	
*Age*	
Mean (SD)	34.9 (10.6)
Range	18.6–56.4
Male/female	37/23
*Race (%)*	
Asian	3 (5 %)
Black	11 (18.3 %)
White	42 (70 %)
Other	4 (6.7 %)
**Operative data**	
*Procedure (%)*	
ACL reconstruction	59 (98.3 %)
ACL reconstruction with medial meniscus repair	1 (1.7 %)
*Location of interference screw (%)*	
Femur	29 (48.3 %)
Tibia	31 (51.7 %)
*Graft characteristics*	
Bone-tendon-bone	22 (36.7 %)
Allograft	18 (30.0 %)
Soft tissue	15 (25.0 %)
Bone-tendon-bone and allograft	3 (5.0 %)
Quadriceps tendon	1 (1.7 %)
Hamstring autograft	1 (1.7 %)
*Anesthesia type (%)*	
General	34 (56.7 %)
Regional block	26 (43.3 %)

ACL, anterior cruciate ligament; SD, standard deviation.

There was a 100 % rate of graft fixation survival at 6 months (54/54; 95 % CI: 100-100) and 1 year (50/50; 95 % CI: 100-100). One patient experienced a complete tear of the ACL 5 months following index surgery, resulting in graft survival rates of 98.1 % (53/54; 95 % CI: 94.6–100) at 6 months and 98.0 % (49/50; 95 % CI: 94.1–100) at 1 year.

Significant improvements (p < .0001) were observed between baseline and 6 months for the majority of patient-reported outcomes, and were maintained out to 1-year follow up (Table 2). EQ-5D VAS scores at 6 months were not significant over baseline, but were so at 1 year. There was no significant difference over baseline in the IKDC sub-scale of symptoms. As a part of the Tegner Activity form, patients were asked to retrospectively rate their knee before injury, which resulted in a mean score of 7.1 (SD, 2.3).

**Table 2:** Patient-reported outcomes at baseline, 6 months, and 1 year.^a^

	Baseline (n = 54)	6 months (n = 50)	1 year (n = 54)
IKDC overall			
Mean (SD)	46.6 (11.5)	62.5 (12.6)	68.1 (10.4)
P-value^b^	–	p < .0001	p < .0001
**IKDC symptom**			
Mean (SD)	17.3 (3.9)	16.2 (3.5)	17.3 (3.2)
P-value	–	p = .1432	p = 1.000
**IKDC sport**			
Mean (SD)	19.2 (7.7)	30.4 (8.4)	33.7 (6.2)
P-value	–	p < .0001	p < .0001
**IKDC function**			
Mean (SD)	4.1 (2.4)	7.7 (2.1)	8.3 (2.0)
P-value	–	p < .0001	p < .0001
**Tegner**			
Mean (SD)	2.4 (1.6)	4.6 (2.4)	5.4 (2.0)
P-value	–	p < .0001	p < .0001
**Lysholm**			
Mean (SD)	54.0 (21.9)	83.5 (16.0)	87.8 (13.2)
P-value	–	p < .0001	p < .0001
**EQ-5D-5L index**			
Mean (SD)	0.76 (0.16)	0.89 (0.13)	0.90 (0.10)
P-value	–	p < .0001	p < .0001
**EQ-5D VAS**			
Mean (SD)	78.4 (16.2)	82.2 (14.0)	84.7 (11.7)
P-value	–	p = .324	p = .0047

EQ-5D-5L,EQ (EuroQuol)-5D-5L; IKDC, International Knee Documentation Committee; SD,standard deviation; VAS,visual analogue scale.

a Last Observation Carried Forward applied to account for missing data.

b P-values are a comparison with baseline values.

Nine patients (15.0 %) experienced serious adverse events during the course of the study. In 7 patients, these adverse events were considered unrelated to the device/procedure, and included right knee joint effusion and arthrofibrosis 4 months later, which successfully resolved with arthroscopic loose body removal and synovectomy; left knee arthrofibrosis, chondromalacia, and the presence of loose bodies, which resolved following treatment including manipulation under anesthesia and chondroplasty; left knee pain 7 months after surgery; a left torn meniscus in the contralateral knee 11 months following initial surgery; left elbow pain and epicondylitis and the previously reported ACL tear; anaphylaxis as a result of food allergy; and soft-tissue mass of the finger.

In addition to the ACL tear, serious adverse events related to the device/procedure included one patient who experienced pulmonary embolism and deep vein thrombosis approximately 1 month following surgery (rated as definitely procedure-related) and another patient who experienced a patellar fracture (rated as possibly related to the surgical procedure).

## 4 Discussion

**Fig. 2:**
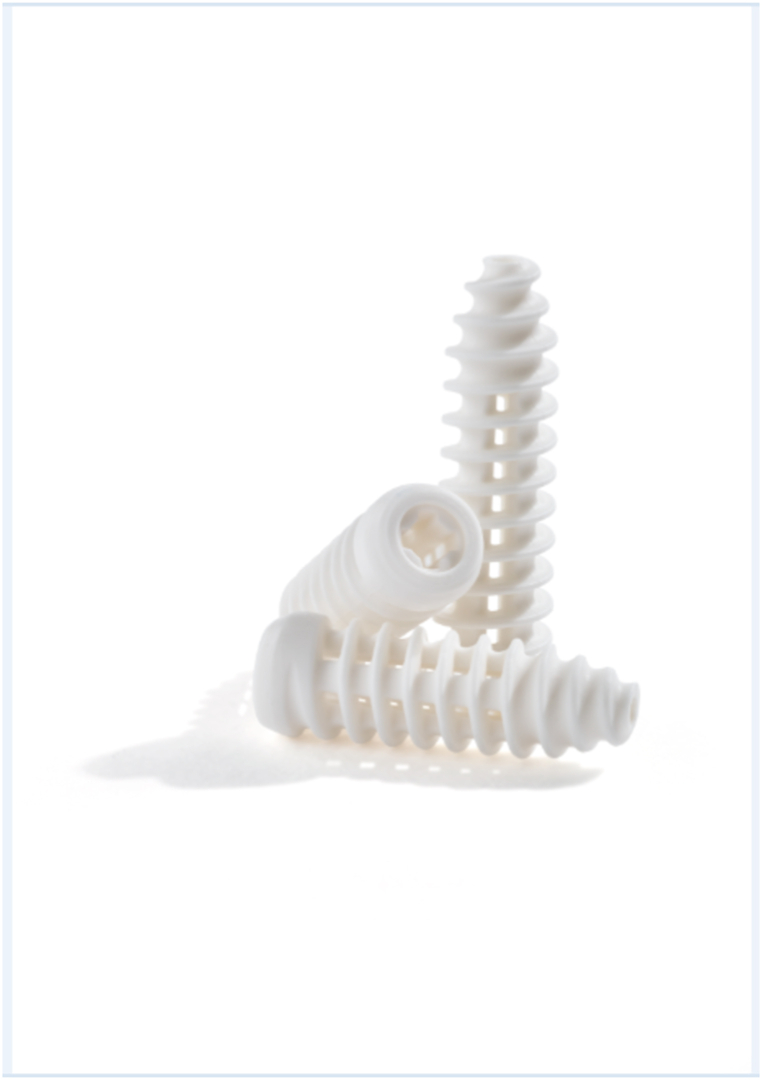
Bioinductive interference screw with open architecture design.

The most important finding from this prospective, multi-center study is that this novel biocomposite interference screw (Fig. 2) resulted in no reports of graft fixation failure at 1 year. Furthermore, patients experienced significant improvements in measurements of postoperative mobility, function, and pain, and none of the serious adverse events encountered were unanticipated or associated with the interference screw.

The failure to obtain sufficient graft fixation at the tibia or femur can lead to incomplete graft fixation and an increased risk of failure [14]. This is particularly important during the initial postoperative period when stress and associated micromotions can impair proper graft integration [15]. The lack of graft fixation failures reported in the current analysis is therefore a promising initial finding, especially as it was obtained across a variety of graft types secured at both the tibia and femur. The graft fixation rate compares favorably with rates reported in the literature with other fixation methods, including bioabsorbable screws, although the differing follow-up periods employed makes for imprecise comparison [[16], [17], [18]].

These results provide the first clinical evidence with this device, and support the underlying hypothesis that its design would facilitate robust graft fixation during the initial postoperative period. Interference screws facilitate joint-adjacent fixation by compressing the graft against the tunnel wall, potentially limiting both graft-tunnel movement and the entry of synovial fluid within the tunnel. In recent years, refinements to the underlying materials and designs have been made to further improve the performance of these bioabsorbable interference screws. In vitro data suggest that the bioabsorbable polymer PLGA mixed with the bioceramic filler β‐TCP makes for an ideal material combination for achieving graft stability, accelerated degradation, and the promotion of bone ingrowth when used in interference screws in ACL reconstruction [19].

Results from the current analysis indicate a significant improvement over baseline was achieved by 6 months in the majority of patient-reported outcomes. The wide variety of surgical techniques, fixation methods, and patient populations reported in the literature for ACL reconstructions make a comparison of studies difficult. However, these results are generally in line with those reported from other recent studies (within the last 5 years) with 1 year or less follow up and using the same patient-reported outcomes [[20], [21], [22], [23], [24]].

The majority of primary ACL tears occur in those between the ages of 17 and 35 years [25], and patient expectations following ACL reconstruction are high [26]. Subjective functional improvements can be evident as early as 2 months following ACL reconstruction [27], but continue to improve throughout the first postoperative year and beyond [27,28]. It is therefore possible that the clinical outcomes reported here may represent underestimates.

Although occurring at relatively low rates, authors have reported complications with biodegradable interference screws including tunnel widening, prolonged knee effusion, cyst formation, and screw migration and breakage [29,30]. A recent network meta-analysis has found that although graft fixation devices used in ACL reconstruction may produce comparable clinical outcomes (i.e., Lysholm, IKDC, Tegner), they have different safety profiles that must be considered [15]. Therefore, it is important for those selecting amongst different classes of devices and surgical methods to confirm that robust fixation can be achieved without accompanying complications. Results from the current study indicate a favorable safety profile with this device, with none of the serious adverse events encountered, considered to be related to the study device. This is consistent with recent reports indicating decreased complication rates with newer materials used for bioabsorbable interference screws, such as β-TCP [31].

The current study has several limitations that should be noted when considering its results. Firstly, the 1-year follow-up duration may be insufficient for capturing the risk of subsequent reinjury, which has been shown to occur primarily within the first 2 years following initial ACL reconstruction [32]. Secondly, the study protocol did not account for when ACL reconstruction was performed following the initial tear. There is ongoing debate surrounding the optimal timing for ACL reconstruction with regards to both revision and clinical outcomes [[33], [34], [35]], and isolating and analyzing this factor in our cohort would have provided data of additional interest to surgeons. Thirdly, to be reflective of real-world practice, the investigators employed a variety of graft types. Clinical outcomes were reported as being mixed depending on whether patients receive autografts or allografts [36]. However, the size of the cohort prevented a sufficiently powered subgroup analysis to determine whether graft selection was a significant predictor of clinical outcomes. Finally, the inclusion of imaging data would have been of interest for determining the course of absorption of the biocomposite interference screw, but was beyond the scope of the current study. Future studies should also consider the potential for intraoperative parameters, such as graft location, pretension, and fixation angle, to effect surgical outcomes.

## 5 Conclusion

In conclusion, this prospective, multi-center study indicates that the biocomposite interference screw, described here, has a favorable safety and efficacy profile at 1 year, with no failures of graft fixation noted during that time. Further follow-up will be needed to determine whether these effects are maintained beyond the initial postoperative period.

## Ethics statement

This study was reviewed and approved by the Western Institutional Review Board (Puyallup, Washington) with the approval number: 20,172,619. All participants/patients (or their proxies/legal guardians) provided informed consent to participate in the study. All participants/patients (or their proxies/legal guardians) provided informed consent for the publication of their anonymised case details and images.

## Data availability statement

Data associated with this study has not been deposited into a publicly available repository as the authors do not have permission to share this data.

## CRediT authorship contribution statement

**Anup Shah:** Writing – review & editing, Writing – original draft, Investigation, Formal analysis, Data curation. **Geoffrey Van Thiel:** Writing – review & editing, Writing – original draft, Investigation, Formal analysis, Data curation.

## Declaration of competing interest

The authors declare the following financial interests/personal relationships which may be considered as potential competing interests:

Smith and Nephew provided support for this manuscript (medical writing and article processing charges).

Dr Van Thiel - Vericel and Smith and Nephew (Consultant/Speaking/Teaching).
