# Supplementary material for: Anterior cruciate ligament reconstruction with a biocomposite interference screw maintains graft fixation survival and improves clinical outcomes at 1 year: A multicenter prospective case series
Source: Heliyon. 2023 Oct 12;9(10):e20921. doi: 10.1016/j.heliyon.2023.e20921 (PMC10585286; doi:10.1016/j.heliyon.2023.e20921)
Supplement: Multimedia component 1 [file mmc1.pdf]

2000 IKDC SUBJECTIVE KNEE EVALUATION FORM

Name: ______ ______ **Date:** ______

First Last

Physician: ______ **Date of Injury:** ______

SYMPTOMS*:

*Grade symptoms at the highest activity level at which you think you could function without significant symptoms, even if you are not actually performing activities at this level.

1.What is the highest level of activity that you can perform without significant knee pain?

- ◯ Very strenuous activities like jumping or pivoting as in basketball or soccer
- ◯ Strenuous activities like heavy physical work, skiing or tennis
- ◯ Moderate activities like moderate physical work, running or jogging
- ◯ Light activities like walking, housework or yard work
- ◯ Unable to perform any of the above activities due to knee pain

2.During the past 4 weeks, or since your injury, how often have you had pain?

	0	1	2	3	4	5	6	7	8	9	10	
Never	◯	◯	◯	◯	◯	◯	◯	◯	◯	◯	◯	Constant

3.If you have pain, how severe is it?

	0	1	2	3	4	5	6	7	8	9	10	
No pain	◯	◯	◯	◯	◯	◯	◯	◯	◯	◯	◯	Worst pain imaginable

4.During the past 4 weeks, or since your injury, how stiff or swollen was your knee?

- ◯ Not at all
- ◯ Mildly
- ◯ Moderately
- ◯ Very
- ◯ Extremely

5.What is the highest level of activity you can perform without significant swelling in your knee?

- ◯ Very strenuous activities like jumping or pivoting as in basketball or soccer
- ◯ Strenuous activities like heavy physical work, skiing or tennis
- ◯ Moderate activities like moderate physical work, running or jogging
- ◯ Light activities like walking, housework or yard work
- ◯ Unable to perform any of the above activities due to knee swelling

6.During the past 4 weeks, or since your injury, did your knee lock or catch?

◯ Yes ◯ No

7.What is the highest level of activity you can perform without significant giving way in your knee?

- ◯ Very strenuous activities like jumping or pivoting as in basketball or soccer
- ◯ Strenuous activities like heavy physical work, skiing or tennis
- ◯ Moderate activities like moderate physical work, running or jogging
- ◯ Light activities like walking, housework or yard work
- ◯ Unable to perform any of the above activities due to giving way of the knee

SPORTS ACTIVITIES:

8.What is the highest level of activity you can participate in on a regular basis?

- ◯ Very strenuous activities like jumping or pivoting as in basketball or soccer
- ◯ Strenuous activities like heavy physical work, skiing or tennis
- ◯ Moderate activities like moderate physical work, running or jogging
- ◯ Light activities like walking, housework or yard work
- ◯ Unable to perform any of the above activities due to knee

9.How does your knee affect your ability to:

		Not difficult at all	Minimally difficult	Moderately Difficult	Extremely difficult	Unable to do
a.	Go up stairs	◯	◯	◯	◯	◯
b.	Go down stairs	◯	◯	◯	◯	◯
c.	Kneel on the front of your knee	◯	◯	◯	◯	◯
d.	Squat	◯	◯	◯	◯	◯
e.	Sit with your knee bent	◯	◯	◯	◯	◯
f.	Rise from a chair	◯	◯	◯	◯	◯
g.	Run straight ahead	◯	◯	◯	◯	◯
h.	Jump and land on your involved leg	◯	◯	◯	◯	◯
i.	Stop and start quickly	◯	◯	◯	◯	◯

FUNCTION:

10. How would you rate the function of your knee on a scale of 0 to 10 with 10 being normal, excellent function and 0 being the inability to perform any of your usual daily activities which may include sports?

FUNCTION PRIOR TO YOUR KNEE INJURY:

	0	1	2	3	4	5	6	7	8	9	10	
Couldn't perform daily activities	◯	◯	◯	◯	◯	◯	◯	◯	◯	◯	◯	No limitation in daily activities

CURRENT FUNCTION OF YOUR KNEE:

	0	1	2	3	4	5	6	7	8	9	10	
Cannot perform daily activities	◯	◯	◯	◯	◯	◯	◯	◯	◯	◯	◯	No limitation in daily activities

IKDC Score

Print Form

Submit
