# Supplementary material for: Anterior cruciate ligament reconstruction with a biocomposite interference screw maintains graft fixation survival and improves clinical outcomes at 1 year: A multicenter prospective case series
Source: Heliyon. 2023 Oct 12;9(10):e20921. doi: 10.1016/j.heliyon.2023.e20921 (PMC10585286; doi:10.1016/j.heliyon.2023.e20921)
Supplement: Multimedia component 2 [file mmc2.pdf]

TEGNER ACTIVITY LEVEL SCALE

Please indicate in the spaces below the HIGHEST level of activity that you participated in BEFORE YOUR INJURY and the highest level you are able to participate in CURRENTLY.

BEFORE INJURY: Level__________ CURRENT: Level___________

**Level 10**	**Competitive sports- soccer, football, rugby (national elite)**
**Level 9**	**Competitive sports- soccer, football, rugby (lower divisions), ice hockey, wrestling, gymnastics, basketball**
**Level 8**	**Competitive sports- racquetball or bandy, squash or badminton, track and field athletics (jumping, etc.), down-hill skiing**
**Level 7**	**Competitive sports- tennis, running, motorcars speedway, handball**<br><br>**Recreational sports- soccer, football, rugby, bandy, ice hockey, basketball, squash, racquetball, running**
**Level 6**	**Recreational sports- tennis and badminton, handball, racquetball, down-hill skiing, jogging at least 5 times per week**
**Level 5**	**Work- heavy labor (construction, etc.)**<br><br>**Competitive sports- cycling, cross-country skiing,**<br><br>**Recreational sports- jogging on uneven ground at least twice weekly**
**Level 4**	**Work- moderately heavy labor (e.g. truck driving, etc.)**
**Level 3**	**Work- light labor (nursing, etc.)**
**Level 2**	**Work- light labor**<br><br>**Walking on uneven ground possible, but impossible to back pack or hike**
**Level 1**	**Work- sedentary (secretarial, etc.)**
**Level 0**	**Sick leave or disability pension because of knee problems**

Y Tegner and J Lysolm. ***Rating Systems in the Evaluation of Knee Ligament Injuries.*** **Clinical Orthopedics and Related Research. Vol. 198: 43-49, 1985.**

SURGI CAL HI STORY

Have you had any additional surgeries to your knee other than those performed by Dr. Stone?

Yes / No

If Yes:

What procedure(s) were performed? ____

When was the surgery performed?____

Who performed the surgery?____
