# Supplementary material for: Anterior cruciate ligament reconstruction with a biocomposite interference screw maintains graft fixation survival and improves clinical outcomes at 1 year: A multicenter prospective case series
Source: Heliyon. 2023 Oct 12;9(10):e20921. doi: 10.1016/j.heliyon.2023.e20921 (PMC10585286; doi:10.1016/j.heliyon.2023.e20921)
Supplement: Multimedia component 3 [file mmc3.pdf]

LYSHOLM KNEE SCORING SCALE

Instructions: Below are common complaints which people frequently have with their knee problems. Please check the statement which best describes your condition.

I. LIMP:
- _____ I have no limp when I walk. (5)
- _____ I have a slight or periodical limp when I walk. (3)
- _____ I have a severe and constant limp when I walk. (0)

II. USING CANE OR CRUTCHES
- _____ I do not use a cane or crutches. (5)
- _____ I use a cane or crutches with some weight-bearing. (2)
- _____ Putting weight on my hurt leg is impossible. (0)

III. LOCKING SENSATION IN THE KNEE
- _____ I have no locking and no catching sensations in my knee. (15)
- _____ I have catching sensation but no locking sensation in my knee. (10)
- _____ My knee locks occasionally. (6)
- _____ My knee locks frequently. (2)
- _____ My knee feels locked at this moment. (0)

IV. GIVING WAY SENSATION FROM THE KNEE
- ____ My knee never gives way. (25)
- ____ My knee rarely gives way, only during athletics or other vigorous activities. (20)
- ____ My knee frequently gives way during athletics or other vigorous activities, in turn I am unable to participate in these activities. (15)
- ____ My knee occasionally gives way during daily activities. (10)
- ____ My knee often gives way during daily activities. (5)
- ____ My knee gives way every step I take. (0)

V. PAIN:
- ____ I have no pain in my knee. (25)
- ____ I have intermittent or slight pain in my knee during vigorous activities. (20)
- ____ I have marked pain in my knee during vigorous activities. (15)
- ____ I have marked pain in my knee during or after walking more than 1 mile. (10)
- ____ I have marked pain in my knee during or after walking less than 1 mile. (5)
- ____ I have constant pain in my knee. (0)

VI. SWELLING
- ____ I have no swelling in my knee. (10)
- ____ I have swelling in my knee only after vigorous activities. (6)
- ____ I have swelling in my knee after ordinary activities. (2)
- ____ I have swelling constantly in my knee. (0)

VII. CLIMBING STAIRS:
- ____ I have no problems climbing stairs. (10)
- ____ I have slight problems climbing stairs. (6)
- ____ I can climb stairs only one at a time. (2)
- ____ Climbing stairs is impossible for me. (0)

VIII. SQUATTING
- ____ I have no problems squatting. (5)
- ____ I have slight problems squatting. (4)
- ____ I can not squat beyond a 90 degree bend in my knee. (2)
- ____ Squatting is impossible because of my knee. (0)

TOTAL_____/100

INSTRUCTIONS: Please place an X on the line to indicate the amount of pain you have had in your knee(s) the past 24 hours. The scale ranges from "no pain at all" to the "worst possible pain".

RIGHT KNEE ____

no pain worst possible pain

LEFT KNEE ____

no pain worst possible pain
